# Development and preliminary validation of a multimodal instrument for spasticity quantification using a composite index: A pilot study

**DOI:** 10.1371/journal.pone.0348378

**Published:** 2026-05-04

**Authors:** Juan Manuel Rosero Ñañez, Sabrina Schaly, Elizabeth Roldán Gonzalez, María de los Ángeles Urbano Díaz, Julián Antonio Villamarín Muñoz

**Affiliations:** 1 Faculty of Engineering, The University of Sydney, School of Biomedical Engineering, Sydney, Australia; 2 Fundación Universitaria María Cano, Physiotherapy Program, Research Group Importance of Movement of Human Development, Fisioter, Colombia; 3 Southwest State Social Enterprise, Health Care Unit of Mercaderes, Diagnostic Support and Therapeutic Complementation Group, Mercaderes, Colombia; 4 Antonio Nariño University, Faculty of Mechanical, Electronic, and Biomedical Engineering, Popayán, Colombia; Transilvania University of Brasov: Universitatea Transilvania din Brasov, ROMANIA

## Abstract

Spasticity is a frequent neuromuscular impairment associated with cerebral palsy, stroke, and spinal cord injury, commonly assessed using subjective clinical scales. This exploratory pilot study aimed to develop and preliminarily validate a multimodal instrument for the objective quantification and stratification of spasticity in nine individuals (3 female, 6 male) with upper-limb spasticity due to cerebral palsy (n = 5) or stroke (n = 4). A wearable system integrating surface electromyography, inertial measurement units, and force sensing resistors was designed to simultaneously capture muscle activation, joint kinematics, and generalized resistance force during standardized passive mobilizations. Simple indicators six area under the curve-based indicators were derived: force, sEMG, and angular velocity under two conditions (R1, R2) and given distinct weights depending on their contribution. Principal component analysis revealed that three latent components accounted for 83.86% of the total variance observed across participants. Based on these indicators, a Composite Index was constructed using min–max normalization and weighted linear aggregation. Within the pilot study, the Composite Index could differentiate between spasticity severity levels (F = 6.38, p = 0.0327, η² = 0.68), with sEMG activity during slow stretch (AUC sEMG R2) the most influential contributor indicators. The proposed multimodal instrument demonstrates preliminary feasibility as a non-invasive and portable approach for objective spasticity quantification, warranting further validation in larger cohorts.

## Introduction

Spasticity is a multifactorial neuromuscular condition affecting between 30% and 80% of individuals with central nervous system injuries, such as spinal cord trauma, stroke, and cerebral palsy [1–3]. Its pathophysiology is based on disruptions in descending inhibitory mechanisms and alterations in neuronal plasticity, which are hallmarks of upper motor neuron syndromes. Consequently, increased resistance to passive stretch is observed in proportion to the velocity of movement [4,5]. In addition to hypertonia, the condition is associated with exaggerated reflexes, abnormal simultaneous muscle contractions, and involuntary movements factors. These factors impair functional mobility and increase healthcare costs by 18% annually in middle-income countries, according to epidemiological reports [6–8]. In pediatric populations, delayed diagnosis contributes to irreversible musculoskeletal complications such as scoliosis and hip dislocation, present in 68% of children with cerebral palsy [9,10].

Current clinical evaluation of spasticity primarily relies on tools such as the Modified Ashworth Scale (MAS) and the Modified Tardieu Scale (MTS), which are widely used in therapeutic contexts but limited by their subjectivity. MAS rates resistance to passive movement on a scale from zero (normal tone) to four (rigid joint). The MTS assesses two key angles during muscle stretching: the angle of reflex activation (R1) at high speed, and the range of passive motion (R2) at low speed [11–14]. Despite their usefulness, their subjective nature results in reduced inter- and intra-rater variability (k = 0.45–0.62), affecting reproducibility. Multicenter studies report up to 32% variation in botulinum toxin (BoNT) dosing for the same patient evaluated by different clinicians [15–17].

Similarly, qualitative approaches like the Spasm Frequency Scale (SFS) oversimplify the complexity of the condition by using discrete categories (e.g., “occasional” vs. “frequent”) without integrating the interaction between neural (reflex hyperactivity) and non-neural (fibrosis) factors [18]. This issue is particularly relevant in pediatric cases of spastic cerebral palsy, where overlapping spastic and dystonic symptoms often lead to diagnostic errors, as revealed by electromyographic analyses [19,20].

In children with cerebral palsy (CP), assessments faces additional challenges due to maladaptive neural plasticity during development which perpetuates motor deficits into adulthood [21,22]. Hence, tools such as MAS and MTS which depend on the examiner’s perception, demonstrate low precision in complex clinical settings and in the presence of pain or hyperreflexia [23–25]. To address these limitations, emerging technologies have been explored. Surface electromyography (sEMG), for instance, enables differentiation between neural (reflex hyperactivity) and non-neural (tissue stiffness) components of hypertonia [26–28]. A recent study introduced the use of the t¯k parameter (normalized time to peak activation) as a quantitative measure of spasticity, obtained through maximum voluntary contractions. In a study with six post-stroke patients, t¯k showed a correlation of r ≈ 0.96 (p < 0.05) with MAS scores and a mean error below 14% relative to sEMG data. Nevertheless, its application was limited to isometric elbow tests and only two muscles, with manual model configuration [29,30]. Additionally, inertial measurement units (IMUs) have enabled real-time quantification of kinematic variables such as angular velocity and joint displacement. These data, integrated with sEMG recordings, support the development of quantitative spasticity indices such as the Spasticity Assessment Score (SAS), which evinced a strong correlation with the MAS (r = 0.86), albeit with methodological limitations [31,32]. Similarly, wearable devices such as gloves with force sensing resistors (FSRs) have been used to monitor joint torque during pharmacological interventions, though often with fragmented approaches [33,34].

Nonetheless, current technologies used for instrumental spasticity assessment present significant limitations when applied in isolation. Although sEMG is suited for recording muscle electrical activity, it lacks information concerning the movement’s kinematic context, which hinders functional interpretation [35–37]. Meanwhile, IMUs provide detailed information about body segment kinematics, such as acceleration, displacement, or angular velocity, but cannot identify the underlying neuromuscular activation that generates motion. Lastly, FSRs commonly used to register pressure or contact force cannot distinguish whether resistance originates from a reflex nervous system response or from structural tissue alterations such as fibrosis or contracture [38,39]. The absence of coordinated acquisition of the different components of the spastic phenomenon severely limits its comprehensive characterization, especially in complex clinical phenotypes where spasticity and dystonia are common. Likewise, the lack of tools capable of simultaneously capturing kinematic variables, dynamic neuromuscular signals such as sEMG activity, and mechanical manifestations hinders the development of accurate disorder profiles. This delays the implementation of timely and personalized therapeutic interventions.

This study provides support for a multimodal Composite Index (CI) that integrates biomechanical and neuromuscular indicators derived from generalized force (Qj), angular velocity (ω) and sEMG signals. By overcoming the limitations of isolated instrumental approaches, the model integrates biomechanical and neurophysiological information into a single quantitative parameter capable of representing both reflex hyperactivity and passive muscle tissue stiffness. Given the studies sample size, the present study is designed as an exploratory, hypothesis-generating pilot investigation to assess the feasibility and internal coherence of a multimodal composite index.

## Materials and methods

### Participants

The study included nine patients with a clinical diagnosis of muscle spasticity secondary to neurological conditions such as cerebral palsy or stroke. The spasticity level was classified using the MTS, under the supervision of a trained physiotherapist independent from the physiotherapist responsible for conducting the evaluation procedure

Participants were prospectively recruited from the neuromuscular rehabilitation unit of a public healthcare institution in southwestern Colombia between November and December 2023. Inclusion criteria included clinically documented spasticity, age ≥ 18 years, ability to cooperate during the evaluation protocol, and overall medical stability. Exclusion criteria included recent orthopedic surgery (within the past six months), active infections, and the use of muscle relaxants or botulinum toxin within seven days prior to assessment.

Demographic data including sex, age, height, weight, body mass index (BMI), diagnosis, and type of spasticity were collected through a structured interview conducted prior to evaluation and recorded using a standardized study form. These variables are detailed in Table 3. All participants or their legal guardians, when applicable, signed a written informed consent form prior to enrollment.

**Table 3:** Individual participant characteristics (n = 9).

Participant #	Sex	Age (years)	Height (cm)	Weight (kg)	BMI (kg/m^2^)	Diagnosis	Type of Spasticity
1	M	18	140	45	22,96	Cerebral Palsy	MTS (Upper limb)
2	M	21	170	60	20,8	Cerebral Palsy	MTS (Upper limb)
3	M	25	165	55	20,2	Cerebral Palsy	MTS (Upper limb)
4	F	20	150	40	17,8	Cerebral Palsy	MTS (Upper limb)
5	F	23	155	48	20	Cerebral Palsy	MTS (Upper limb)
6	M	30	168	62	22	Stroke	MTS (Upper limb)
7	M	36	170	65	22,5	Stroke	MTS (Upper limb)
8	M	42	160	58	22,7	Stroke	MTS (Upper limb)
9	F	28	150	52	23,1	Stroke	MTS (Upper limb)

The study was approved by the Ethics Committee of the Health Care Unit of Mercaderes (Approval No. 02–2023, Minutes No. 09), and all procedures were carried out in accordance with the ethical principles set forth in the Declaration of Helsinki, the Belmont Report, and the U.S. Code of Federal Regulations.

## Measurement devices

### Surface electromyography (sEMG)

The Myo Armband (Thalmic Labs), a portable device equipped with eight Ag/AgCl electrodes (10 mm diameter, 20 mm inter-electrode distance), was used to record neuromuscular activity from the belly of the biceps brachii on the affected limb. The signal was acquired at 1 kHz, within a *±* 5 mV range and 16-bit resolution. A third-order Butterworth filter (20–500 Hz) was then applied, followed by signal rectification and smoothing using a 100 ms moving window. Synchronization with other devices was achieved via external time stamps.

### Inertial measurement unit (IMU)

The WT901BLECL sensor (WitMotion) was fixed to the distal segment of the forearm to capture kinematic variables. The system integrates a triaxial accelerometer (*±*16 g), triaxial gyroscope (*±*2000^*◦*^/s), and magnetometer (*±*4900 µT), operating at a sampling rate of 100 Hz. Calibration procedures were applied to compensate for drift and offset. The data were used to compute angular velocity (rad/s), linear acceleration (m/s^2^), and joint angles via the Madgwick sensor fusion algorithm.

### Sensorized glove

A custom glove was designed with nine resistive force sensors (FSR 402, Interlink Electronics; range: 0.1–10 N; resolution: *±* 0.05 N) distributed across the distal phalanges and palm of the evaluator’s hand. The FSRs were connected to an Arduino Nano microcontroller (50 Hz) via voltage divider circuits. Each sensor was individually calibrated using reference weights (0.5–10 N) and fitted with a third-order polynomial model (*R*^2^ *>* 0.98) to convert voltage outputs into force values.

### Experimental protocol

An experimental protocol was implemented based on the synchronized integration of *Q*_*j*_, *ω*, and sEMG recordings during standardized passive mobilizations according to the MTS. This approach enabled simultaneous and objective characterization of both mechanical stiffness and neuromuscular excitability associated with spasticity. Signal acquisition was carried out under controlled conditions, applying slow (V1) and fast (V3) stretches sequentially, as established by the MTS.

**Fig 1:**
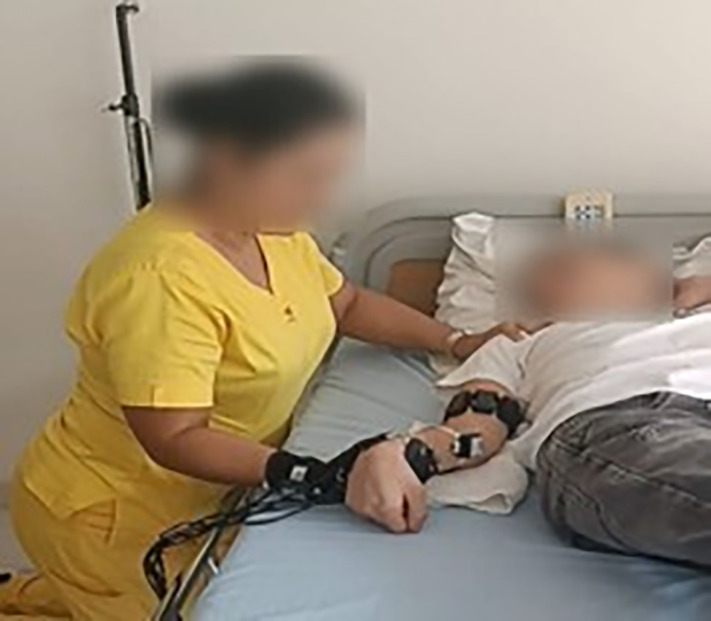
Implementation of the multimodal system during clinical spasticity assessment in the upper limb. The physiotherapist is shown using the FSR-equipped sensorized glove and wears an IMU on the wrist, while the patient is instrumented with an IMU on the distal forearm and sEMG electrodes placed over the biceps brachii muscle.

During the protocol, a trained physiotherapist, equipped with the sensorized glove and IMU sensor performed passive mobilizations of the participant’s elbow, ensuring full joint range under both conditions. The Fig 1 illustrates the practical implementation of the multimodal system in the clinical setting. It shows the evaluator’s position and sensor arrangement: the glove on the physiotherapist’s dominant hand and an IMU sensor on the wrist for monitoring movement velocity an IMU on the patient’s distal forearm, and the sEMG electrodes aligned on the belly of the spastic biceps muscle. This setup enabled simultaneous, non-invasive, and structured capture of the key domains involved in the spastic phenomenon.

### Evaluation procedure

Each participant was evaluated in a seated position, with the shoulder in neutral adduction and the affected elbow initially in maximum passive flexion. All assessments were performed by the same trained physiotherapist equipped with the sensorized glove on the dominant hand, performed standardized passive elbow mobilizations in accordance with MTS guidelines. The protocol included two passive stretch conditions. First, a slow stretch (V1) was applied at a controlled angular velocity of approximately 5◦/s, covering the full joint range from maximal flexion to full extension to identify the R2 angle, corresponding to the passive elongation limit without reflex activation. Then, a fast stretch (V3) was applied at approximately 100◦/s, intended to elicit the muscle’s reflex response and determine the R1 angle, defined as the initial point of spastic resistance or “catch.” To minimize potential evaluation bias, manual clinical classification using the MTS was independently performed by a different physiotherapist, ensuring separation between clinical assessment and instrumented evaluation. Although formal blinding was not implemented during the assessment, the use of objective sensor-based measurements reduces the influence of observer-related bias.

**Fig 2:**
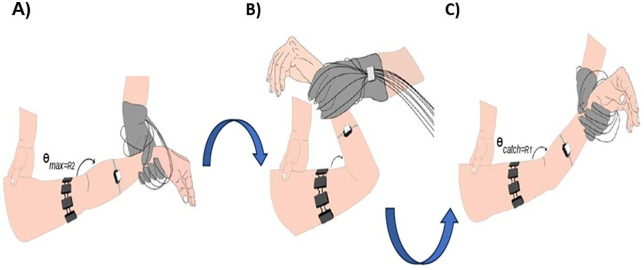
Schematic sequence of the passive elbow evaluation protocol. (A) Slow stretch (V1) from maximal flexion to determine *R*_2_. (B) Transition phase toward joint extension. (C) Fast stretch (V3) to elicit the spastic reflex and determine the *R*_1_ or “catch” angle. The system includes FSR sensors in the evaluator’s glove, an IMU on the forearm, and an sEMG system over the biceps brachii.

Fig 2 shows the procedure sequence. Fig 2A represents the initiation of the slow stretch (V1) from maximum flexion to determine R2. Fig 2B illustrates the transition toward full extension, while Fig 2C shows the fast stretch phase (V3), during which the R1 angle is identified through the spastic reflex.

The order of application was kept constant, with V1 followed by V3, including a 10-second interval between both conditions to reduce the effect of reflex preactivation.

Each condition was repeated three consecutive times to ensure measurement consistency. During fast stretches, the sensorized glove recorded in real time the generalized force Qj applied by the physiotherapist on the patient’s forearm. A sudden increase in this force was interpreted as the onset of spastic resistance (R1). Simultaneously, an IMU fixed on the distal forearm recorded the angular velocity ω, verifying that predefined kinematic criteria were met. Additionally, an sEMG system captured neuromuscular reflex activity in the biceps brachii, complementing the objective analysis with neurophysiological data. This configuration enabled the synchronized acquisition of biomechanical and neuromuscular signals, including generalized force, angular velocity, and neuromuscular excitability. These signals were subsequently processed to derive simple indicators, which were then integrated into the Composite Index.

### Data processing

All collected data were processed offline using *MATLAB R2023a* (MathWorks Inc., Natick, MA, USA) to extract quantitative biomechanical and neurophysiological measures.

Signals from the sensorized glove were converted into force units (Newtons) using third-order polynomial calibration curves fitted with standard weights ranging from 0.5 to 10 N. The signals were then smoothed to eliminate high-frequency noise and obtain a continuous temporal profile of the resistance force exerted during each stretch cycle.

IMU data were filtered by using a 5 Hz low-pass filter and processed to extract elbow joint kinematics, including sagittal plane angular position, instantaneous angular velocity, and acceleration. From these signals, the *R*_2_ angle (maximum elongation without reflex activation) and *R*_1_ angle (onset of the spastic “catch”) were determined according to the MTS protocol. The difference between these angles, expressed as ∆*R* = *R*_2_ *− R*_1_, was used as a differential clinical parameter to characterize spasticity severity.

The *Q*_*j*_ was estimated by using a spatial transformation based on a 3D glove model, incorporating specific angles and distances of each FSR sensor relative to the estimated hand center of mass. This transformation enabled calculation of linear (*Q*_*T*_) and rotational (*Q*_*R*_) force moments for each recorded sample, providing a more accurate biomechanical interpretation of the therapeutic interaction.

Simultaneously, sEMG signals were preprocessed with a third-order bidirectional Butterworth filter with a 20–500 Hz passband. The signals were then rectified and smoothed using a 100 ms moving window to obtain the linear envelope of neuromuscular activation. Activation latency was calculated as the time interval between the onset of the fast stretch (V3 condition) and the significant increase in the EMG signal, along with peak amplitude and the integral following the reflex response, as representations of the excitability of the spastic muscle.

**Fig 3:**
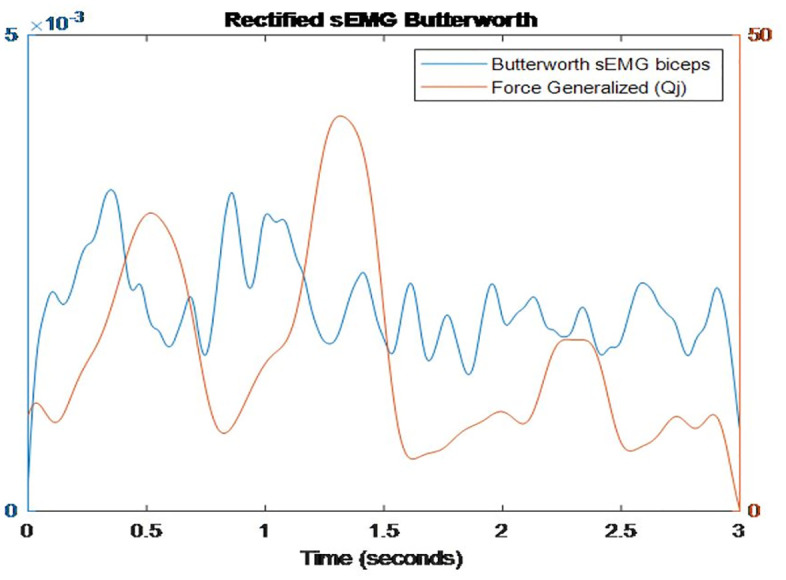
Temporal synchronization between the filtered sEMG signal of the biceps and the generalized force (*Q*_*j*_) during fast stretching.

To integrate sEMG and biomechanical signals, a cross-correlation analysis was performed between the sEMG envelope and the *Q*_*j*_ signal, enabling synchronization and highlighting the reflex delay between mechanical stimulus and muscle activation (see Fig 3).

**Fig 4:**
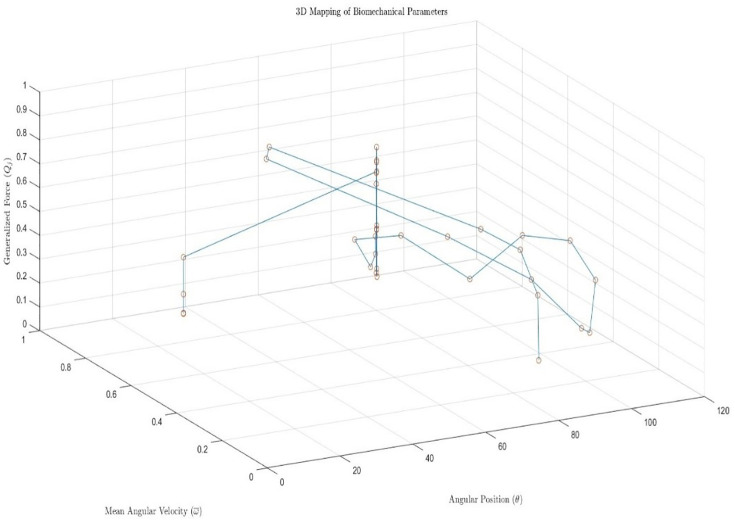
Three-dimensional mapping of the generalized force(*Q*_*j*_), angular position (*θ*), and mean angular velocity (*ω*).

Additionally, a three-dimensional visualization of *Q*_*j*_, angular position (*θ*), and mean angular velocity (*ω*) was generated, as shown in Fig 4. This representation allowed the observation of biomechanical response patterns and relevant kinetic events during passive stretch, supporting the visual validation of the signal processing and subsequent analysis.

### Simple indicators for spasticity assessment

The accuracy and effectiveness of a CI directly depends on the methodological robustness and clinical relevance of the selected simple indicators. These indicators must meet criteria such as high quality, sensitivity to change, adequate sampling frequency, and reliable data availability [40]. In this study, three fundamental domains were identified for spasticity assessment: generalized force (via FSR sensors), joint angular velocity (via IMU), and surface electromyographic activity (sEMG).

For these signals, the Area Under the Curve (AUC) was adopted as the primary approach to quantitatively estimate the neuromuscular response to stretch. AUC provides an integrated measure that combines magnitude and temporal duration, which is particularly relevant in spasticity where both reflex response and passive resistance depend on the intensity and time evolution of the stimulus [41–43]. This approach allows differentiation between passive movement phases, such as the angle at which the spastic reflex appears (R1) and the maximum elongation without reflex (R2), enabling a more precise characterization of muscle dynamics.

The validity of AUC is not limited to clinical contexts. In sports science, it has been used to analyze neuromuscular efficiency during eccentric and concentric contractions, and to assess load management under fatigue or explosive effort conditions [44,45]. To illustrate, it has proven useful in characterizing hamstring and quadriceps performance during sprinting and direction changes, where stride stiffness and ground contact time are influenced by the muscle’s load-absorbing capacity [46].

From an instrumental standpoint, AUC also offers computational advantages. Its implementation is efficient and less susceptible to artifacts or high-frequency noise, as temporal integration smooths out irregularities without requiring aggressive filtering. This makes it a reliable metric in high-variability clinical settings and real-time applications [47].

In this study, six simple AUC-based indicators were computed: two for each signal type (FSR, IMU, sEMG) under R1 and R2 conditions. These variables were normalized by using min-max scaling and Z-score standardization, then subjected to correlation and principal component analysis (PCA) to verify independence and reduce informational redundancy.

For reference, the derived simple indicators are summarized in Table 1, which specifies each metric’s origin and corresponding condition. These indicators serve as the foundation for the CI integration described in the next section.

**Table 1:** Simple indicators for spasticity assessment.

Indicator	Source	Purpose
AUC Force (R1)	FSR (glove)	Reflex resistance
AUC Force (R2)	FSR (glove)	Passive resistance
AUC sEMG (R1)	sEMG (biceps)	Reflex excitability
AUC sEMG (R2)	sEMG (biceps)	Basal activation
AUC Velocity (R1)	IMU (arm)	Fast stretch stimulus
AUC Velocity (R2)	IMU (arm)	Slow stretch stimulus

### Composite Index integration

The CI was developed to synthesize six biomechanical and neuromuscular simple indicators into a single, quantitative measure of spasticity severity. These variables, derived from the area under the curve (AUC) of signals recorded during the MTS protocol and represent different physiological aspects of spasticity, including passive stiffness, reflex activation, and dynamic resistance. Data were acquired using FSR (force), sEMG (muscle activity), and IMU (angular velocity) sensors during both slow (R2) and fast (R1) stretches.

**Fig 5:**
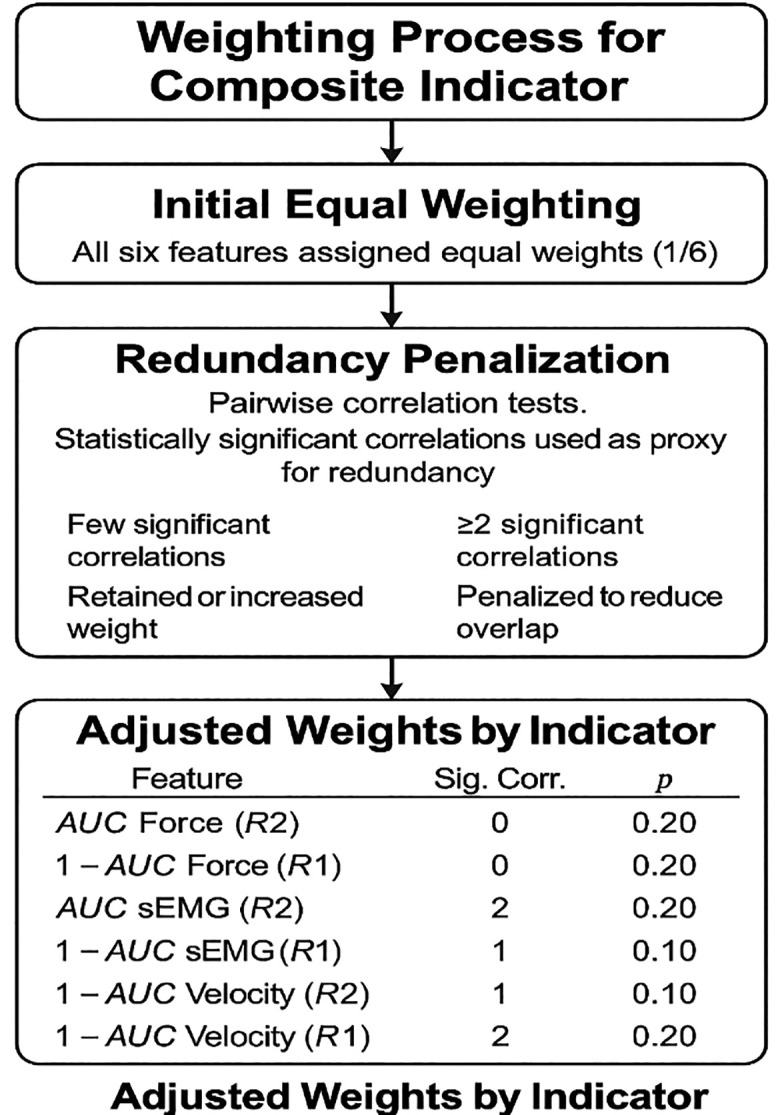
Methodological framework for Composite Index (CI) construction.

A structured four-step methodology was implemented to ensure the internal validity of the CI, encompassing normalization, redundancy analysis, weight refinement and final aggregation, as visually summarized in Fig 5.

(1) Normalization: All six AUC-based indicators were first normalized using min–max scaling to ensure comparability and remove unit dependency. Those where higher raw values indicated lower spasticity (e.g., force at R1) were inverted to maintain interpretability. After normalization, all features were aligned so that higher values reflect greater spasticity.
(2) Independence analysis: To reduce redundancy and preserve the unique contribution of each feature, statistical independence was assessed via pairwise Pearson correlation tests. Table 2 presents the corresponding p-values. A p-value < 0.05 was considered statistically significant.

**Table 2:** P-values between simple indicators.

Metric	AUC Force (R2)	AUC Force (R1)	AUC sEMG (R2)	AUC sEMG (R1)	AUC Vel. (R2)	AUC Vel. (R1)
AUC Force (R2)	1	0.5871	0.9707	0.8673	0.6840	0.4909
AUC Force (R1)	0.5871	1	0.4142	0.4319	0.9315	0.6406
AUC sEMG (R2)	0.9707	0.4142	1	0.0045	0.0033	0.0057
AUC sEMG (R1)	0.8673	0.4319	0.0045	1	0.1636	0.9347
AUC Velocity (R2)	0.6840	0.9315	0.0033	0.1636	1	0.0062
AUC Velocity (R1)	0.4909	0.6406	0.0057	0.9347	0.0062	1

Notably:

- AUC sEMG (R2) was significantly correlated with AUC sEMG (R1) (p = 0.0045) and AUC Velocity (R2) (p = 0.0033).
- AUC sEMG (R1) also showed correlation with AUC Velocity (R1) (p = 0.0062).
- These findings revealed partial redundancy, particularly among the neuromuscular and kinematic simple indicators during fast stretches. In contrast, AUC Force (R1) and AUC Force (R2) showed weaker correlations (p > 0.5), suggesting higher informational independence.

(3) Weight refinement: The weighting strategy used in the CI was designed to be transparent, mathematically justified, and internally valid. Initially, each of the six normalized indicators was assigned an equal weight (1/6), following the principle of uniform importance when no clear hierarchy is established [40]. To quantify the degree of redundancy, we computed a significant correlation count for each indicator. This was defined as the number of pairwise correlations (out of the five possible) with a p-value < 0.05, based on Pearson correlation tests (see Table 2). This count was used as a proxy for feature overlap:
  - Indicators with more significant correlations were considered more redundant and were softly penalized in the final weighting.
  - Indicators with fewer or no significant correlations were deemed more independent and were favored with higher weights.
  - For example, AUC sEMG (R2) exhibited two statistically significant correlations one with AUC sEMG (R1) and one with AUC Velocity (R2) while AUC Force (R1) showed no significant correlation with any other indicator. As a result, the former received a moderate weight, and the latter was prioritized.

This approach reflects a quantitatively motivated heuristic, inspired by methods of correlation-based feature weighting often applied in composite indices and machine learning contexts [48,49]. It minimizes multicollinearity and reinforces the contribution of informationally distinct indicators, thereby improving the robustness and interpretability of the CI. Within this framework, it should be acknowledged that the weighting scheme is derived from statistical relationships identified within a limited sample and is therefore inherently sensitive to cohort specific patterns. Consequently, the assigned weights represent an internally consistent configuration specific to this study, rather than a universally established or generalizable weighting structure. The stability and generalizability of this weighting approach should be further examined through cross-validation and external validation.

The refined weights assigned to each normalized metric were as follows:

*p*_1_ = 0*.*20 (AUC Force (R2))

*p*_2_ = 0*.*20 (1 – AUC Force (R1))

*p*_3_ = 0*.*20 (AUC sEMG (R2))

*p*_4_ = 0*.*10 (1 - AUC sEMG (R1))

*p*_5_ = 0*.*10 (AUC Vel. (R2))

*p*_6_ = 0*.*20 (1 – AUC Vel. (R1))

It is important to note that although AUC sEMG (R2) presented moderate statistical redundancy, it was retained with a balanced weight due to its distinct physiological significance. Specifically, this feature captures baseline muscle activation during slow passive stretch, which reflects non-reflex hypertonia, a clinically relevant manifestation of spasticity not accounted for by reflex mediated variables alone. This sustained neuromuscular activity, often linked to increased muscle tone at rest, plays a crucial role in functional limitations and treatment response. Therefore, the influence of AUC sEMG (R2) in the CI despite its correlation with other variables was preserved to ensure coverage of this important physiological dimension.

(4) Aggregation: Once all six indicators were normalized and assigned refined weights, the CI was calculated through a weighted linear aggregation, a standard method used in multi-criteria assessment frameworks and composite index construction [40,48]. This approach enables a transparent and interpretable synthesis of multidimensional data into a single score that reflects the severity of spasticity.

The final expression of the CI is as follows:

CI = *p*_1_ AUC Force (R2) + *p*_2_*·* (1 *−* AUC Force (R1))

+ *p*_3_*·* AUC sEMG (R2) + *p*_4_*·* (1 *−* AUC sEMG (R1))

+ *p*_5_*·* (1 *−* AUC Vel. (R2)) + *p*_6_*·* (1 *−* AUC Vel. (R1))

This formulation ensures that higher CI values correspond to greater severity of spasticity. The metric integrates contributions from both neural (e.g., reflexive sEMG, force responses) and non-neural (e.g., passive stiffness, baseline tone) sources of resistance. The weighting scheme reflects the statistical independence and informational value of each component, as established in the previous steps.

Linear aggregation was chosen due to its interpretability, additive transparency, and sensitivity preservation meaning that improvements or deteriorations in one component (e.g., reduced AUC sEMG) directly translate to proportional changes in the overall CI, provided all other components remain constant. This property is particularly important for clinical monitoring and longitudinal comparison.

## Results

### Demographic data from the pilot study

Of the nine participants, five were diagnosed with cerebral palsy and four with stroke, all of whom presented upper limb spasticity and were evaluated using the MTS. The mean age was 27 with a standard deviation of 7.9 years. Three of the nine participants were female. The demographic details are outlined in Table 3.

### Multivariate analysis of simple indicators via PCA

To assess redundancy and complementarity among the simple indicators used in the construction of the CI, principal component analysis (PCA) was performed. This procedure enabled the identification of latent dimensions that explain most of the variance in the dataset and allowed visualization of the metric distribution in a reduced three-dimensional space.

**Fig 6:**
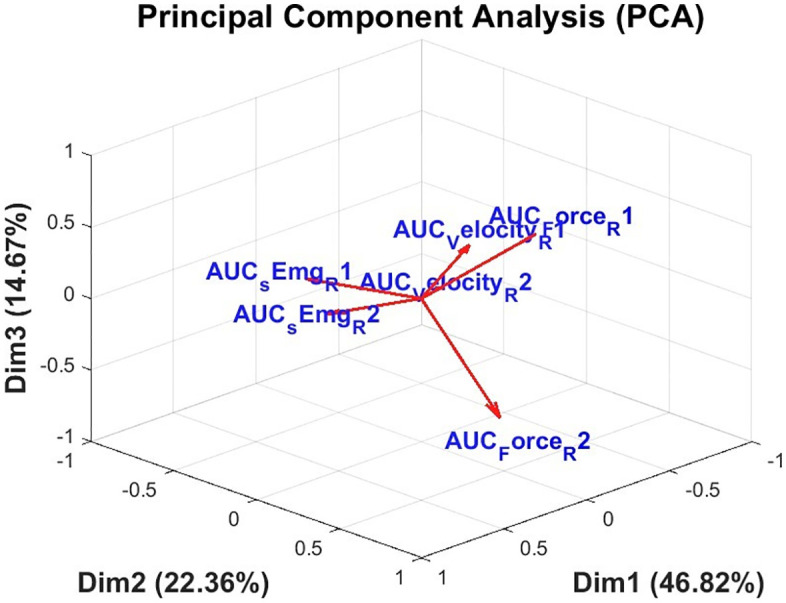
Three-dimensional representation of the principal component analysis (PCA) applied to the normalized indicators. The three dimensions explain 83.85% of the total variance.

Fig 6 shows the 3D biplot resulting from the PCA applied to the six AUC simple indicators, corresponding to generalized force, surface electromyography, and angular velocity under the R1 and R2 conditions of the MTS. The first three principal components explained a combined 83.85% of the total variance: 46.82% for the first dimension (Dim1), 22.36% for the second (Dim2), and 14.67% for the third (Dim3).

The orientation of the vectors in the multivariate space provides insight into the functional grouping of the indicators. The variables AUC Velocity (R1) and AUC Velocity (R2) had marked negative projections on Dim1, aligned with AUC Force (R1), indicating an association between reflex mechanical response and angular velocity patterns. In contrast, AUC sEMG (R2) and AUC sEMG (R1) were grouped in the opposite direction, emphasizing their distinct contribution to neuromuscular activation.

Notably, AUC Force (R2) showed a high loading on Dim3, with a value of 0.656, as shown in the factor loading matrix in Table 4. This indicates that the variable captures a distinct dimension of variability, supporting its inclusion as a non-redundant metric in the CI synthesis.

**Table 4:** Factor loading matrix for the simple indicators.

Metric	Component 1	Component 2	Component 3
AUC Force (R2)	0.067	0.598	0.656
AUC Force (R1)	−0.186	0.588	−0.668
AUC sEMG (R2)	0.557	−0.073	−0.078
AUC sEMG (R1)	0.407	−0.368	−0.17
AUC Velocity (R2)	−0.49	−0.356	0.26
AUC Velocity (R1)	−0.495	−0.172	−0.145

### Sensitivity analysis

Validation of the proposed system was conducted on a cohort of patients with clinically diagnosed spasticity by analyzing both the simple indicators and the CI to evaluate sensitivity, relevance, and discrimination capability. The aim was to determine each metric’s contribution to CI variability, thereby enhancing clinical interpretability of spasticity.

**Fig 7:**
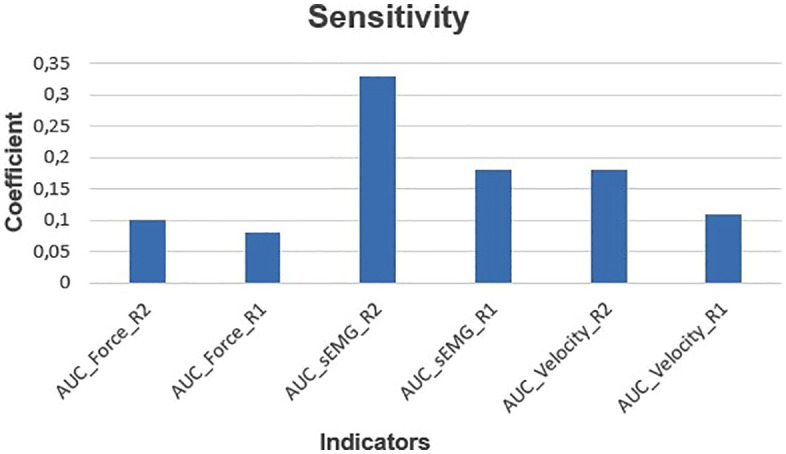
Sensitivity coefficients for each simple indicator contributing to the CI: AUC Force (R2), AUC Force (R1), AUC sEMG (R2), AUC sEMG (R1), AUC Velocity (R2), and AUC Velocity (R1).

Fig 7 shows the analysis estimates of each metric’s relative impact on total index variability, reflecting its discriminative power.

Results indicate that AUC sEMG (R2) has the highest sensitivity coefficient (*≈* 0*.*33), making it the most influential metric in characterizing spastic behavior. Similarly, AUC sEMG (R1) and AUC Velocity (R2) also displayed relatively high sensitivity values, suggesting that both early reflex activation and angular velocity during full stretch are relevant in objectively describing spasticity.

Conversely, AUC Force (R1) showed the lowest sensitivity value, suggesting a reduced influence on the CI. This may be attributed to the tendency of spasticity to manifest more intensely at higher elongation and velocity conditions rather than during initial resistance.

**Fig 8:**
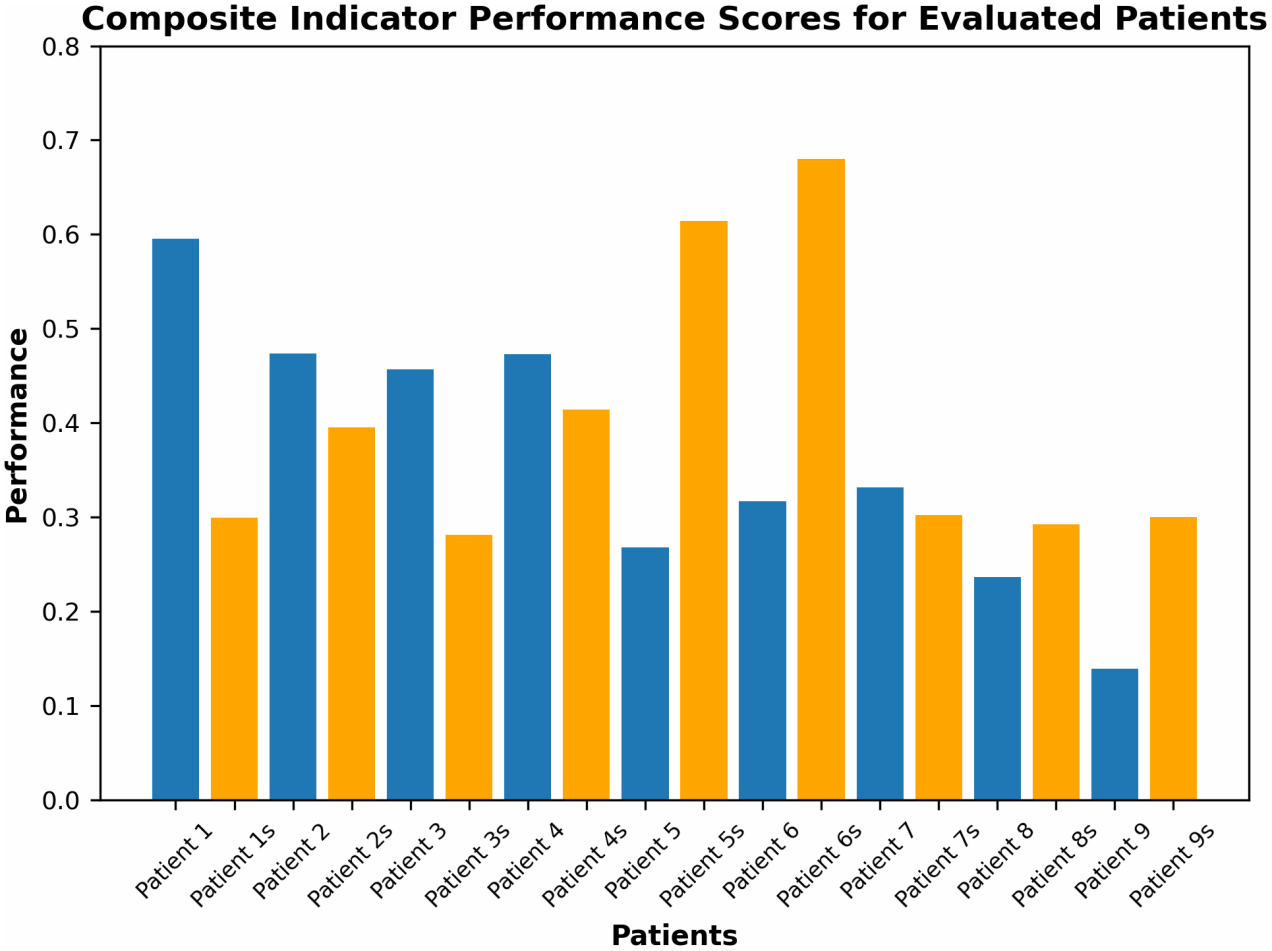
Composite Index values for evaluated patients. The suffix “s” denotes the contralateral non-spastic side.

Fig 8 presents individual CI values for each evaluated subject. Each patient has two records: one for the spastic limb and one for the contralateral side, identified with the suffix “s.” This coding enables intra-individual comparison of neuromuscular behavior between limbs, facilitating detection of spastic asymmetries and possible compensatory mechanisms.

Considerable variability in CI values was observed across participants. For example, patients P1 and P7 exhibited high CI values on the spastic side, indicating greater passive resistance and heightened reflex activation, typical of increased muscle tone. In contrast, patients P9 and P8 showed lower CI values, suggesting either lower spasticity or effective neuromuscular adaptation.

In most cases, significant differences were noted between body sides, confirming the CI’s utility in detecting unilateral alterations characteristic of neurological lesions.

### Correlation analysis between simple indicators and the Modified Tardieu Scale

**Fig 9:**
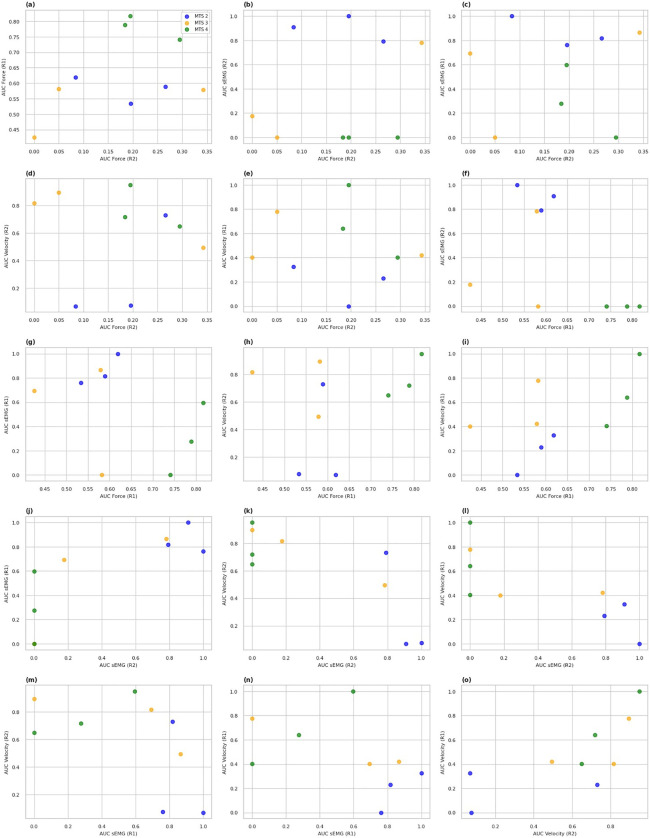
Scatter plots between pairs of simple indicators (AUC values for force, velocity and sEMG) for the spastic limb. Key findings from the analysis include.

To assess the relationship between the biomechanical and neurophysiological simple indicators obtained during the R1 and R2 conditions of the assessment protocol, a cross-correlation analysis was conducted. Fig 9 shows the scatter plots for all pairwise combinations of AUC values in generalized force, angular velocity, and surface EMG, coded by MTS level.

- AUC Force (R2) vs. AUC sEMG (R2): A moderate positive correlation was observed (r = 0.58), indicating that increased reflex activation (sEMG) is associated with greater passive resistance during rapid stretch.
- AUC Force (R2) vs. AUC sEMG (R1): No significant correlation was found, suggesting that low-speed neuromuscular activity does not directly predict passive resistance under rapid conditions.
- AUC Force (R2) vs. AUC Velocity (R2): A moderate negative correlation was found (r = −0.46), indicating that higher angular stretch velocities are associated with lower measured resistance.
- AUC Velocity (R2) vs. AUC sEMG (R2): A moderate inverse trend was noted (r = −0.48), suggesting that higher velocities may correspond to decreased reflex activity.
- AUC sEMG (R2) vs. AUC sEMG (R1): High consistency in neuromuscular response was observed between fast and slow stretches (r = 0.81, p < 0.01).

Combinations such as AUC Force (R1) vs. AUC Velocity (R2) and AUC sEMG (R1) vs. AUC Velocity (R2) showed no significant correlations, suggesting that each metric captures complementary aspects of spasticity.

Table 5 summarizes the ANOVA results comparing CI scores across clinically stratified spasticity groups. The between-group sum of squares was 0.11, the within-group (residual) sum was 0.05, yielding a total variance of 0.16. The resulting F-statistic was 6.38 with a corresponding p-value of 0.03, indicating a statistically significant difference between group means (p < 0.05).

**Table 5:** One-way ANOVA results applied to the CI.

Source	SS	Df	MS	F-stat/ P- value
Between Groups	0,11	2	0,05	F = 6.38, P = 0,03
Within Groups	0,05	6	0,00	–
Total	0,16	8	–	–

This confirms that the CI reflects not only individual biomechanical and neurophysiological characteristics but is also sensitive to clinical classification of spasticity. This discrimination is attributable to the influence of key contributing indicators identified earlier, such as AUC sEMG (R2), AUC sEMG (R1), and AUC Velocity (R2), which showed high sensitivity in the integration model.

**Fig 10:**
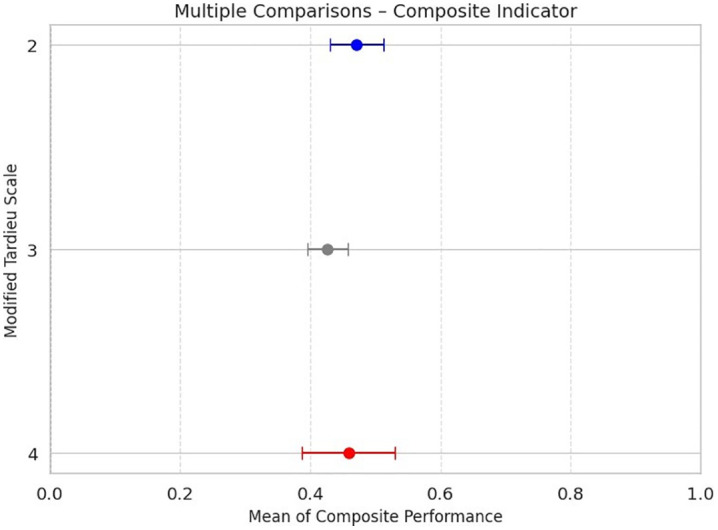
Descriptive comparison of composite performance (*I*_*d*_) across clinical levels of the MTS, considering only body regions affected by muscle tone abnormalities. Each point indicates the mean for levels 2 (blue), 3 (gray), and 4 (red), with corresponding standard error of the mean (SEM).

Fig 10 shows the descriptive comparison of composite performance scores (*I*_*d*_) across clinical spasticity levels based on body regions affected by abnormal tone, as per the MTS. In this horizontal error-bar plot, each point represents the mean *I*_*d*_ for MTS levels 2, 3, and 4, with standard error of the mean (SEM), facilitating intra-group variability interpretation.

Results reveal that the highest average Id occurred in level 2 spasticity (mean = 0.47, 95% Confidence Interval: 0.39–0.55), followed by level 4 (mean = 0.45, 95% Confidence Interval: 0.31–0.60), and level 3 (mean = 0.42, 95% Confidence Interval: 0.36–0.48). This trend suggests that in muscle tone–affected regions, lower clinical spasticity grades are associated with better biomechanical and neuromuscular performance.

The observed effect size (η² = 0.68) indicates a strong association between Composite Index scores and spasticity severity, with 68% of the total variance explained by differences between spasticity levels. While the statistical analysis yielded a significant F-value (p = 0.03), the small sample size may limit the precision of variance estimates. A post hoc power analysis based on the observed η² suggests adequate power to detect large effects; however, the limited number of participants per group may increase the risk of a Type II error failing to identify smaller, yet clinically meaningful differences

While mean values for levels 2 and 4 are close, the wider dispersion in level 4 may reflect greater functional heterogeneity among patients with severe spasticity. These findings highlight the CI’s capacity to sensitively reflect functional differences associated with spastic tone severity.

## Discussion

This pilot study presents a preliminary, multimodal Composite Index designed to quantitatively characterize spasticity by integrating synchronized force, kinematic, and electromyographic signals acquired during standardized passive stretches. The current findings demonstrate the feasibility and internal coherence of combining multiple physiological domains into a single composite index aligned with the Modified Tardieu Scale framework consistent with findings reported using portable technologies and intelligent algorithms for spasticity evaluation [50]. Park et al. also demonstrated the feasibility of training artificial neural networks to emulate clinical spasticity assessment using the MAS, underscoring the potential of machine learning tools in this field [51]. These previous findings support the robustness of the multimodal approach proposed here.

Within this small pilot study, the CI could distinguish between the reflex and non-reflex components of hypertonia. This distinction, which is not explicitly captured by conventional ordinal scales such as the MAS, reflects current neurophysiological understanding of spasticity as a multifactorial phenomenon. Within the present dataset, sustained neuromuscular activity during slow stretch, reflected by AUC sEMG (R2), emerged as a prominent contributor to index variability, suggesting that baseline muscle activation may play an important role in spasticity characterization. However, this differentiation capability must be further validated in larger and more heterogeneous cohorts to confirm its generalizability and diagnostic utility across diverse spasticity profiles. This approach aligns with prior studies: Zhang et al. quantified spasticity by combining EMG and motion data into a unified metric [52], while Bar-On et al. integrated multidimensional signals (e.g., joint torque and EMG) to assess the spastic response in children with cerebral palsy, objectively capturing reflex phenomena such as the “catch” by comparing muscle activity and joint resistance at different velocities [53].

The pilot study CI distinguishes the “catch” reflex response during rapid movements from passive resistance at low velocity. The correlation observed between the CI and MTS further supports its future utility as an objective measure. Unlike the MAS, the MTS explicitly differentiates between the angle of rapid reaction (R1) and full passive range of motion (R2), a distinction structurally embedded in our CI. This conceptual correspondence likely contributed to the strong quantitative agreement, consistent with studies that highlight MTS’s superiority in capturing the velocity-dependent nature of spasticity, as demonstrated by Banky et al. using inertial sensors [54].

The multimodal system builds on previous findings. Kim et al. introduced a method based solely on kinematic data from inertial sensors to assess elbow spasticity, proving useful in classifying muscle tone using purely biomechanical inputs [50]. Likewise, Zhang et al. demonstrated that combining EMG and kinematic data significantly improves the objective assessment of spasticity [52]. The CI reported here leverages the complementarity of multiple sensing modalities (electromyographic data, capturing muscle reflex hyperexcitability, and inertial sensors) to provide a more comprehensive measurement than those derived from a single signal source.

Compared with complex biomechanical models, the CI offers additional advantages in computational simplicity and portability. Ang et al. proposed an upper limb spasticity model with strong alignment to MAS scores [55], highlighting the potential of biomechanical modeling. While effective, such systems often rely on advanced equipment and processing pipelines, which may not be feasible in all clinical settings. The present study prioritizes simplicity and accessibility, using wearable sensors suitable for both clinical and community-based applications. In contrast, the novel CI employs low-cost portable sensors and accessible algorithms, enhancing suitability for both clinical and community-based environments.

Other recent instrumental strategies also underscore the need to enhance objectivity in spasticity assessment. For instance, Choi et al. implemented visual biofeedback mechanisms in IMU-based systems to support MTS assessment in the lower limbs, enhancing inter-rater reliability [56]. Similarly, Jonnalagedda et al. developed an instrumented glove for hand spasticity assessment, showing that portable instrumentation can enhance objectivity [57]. However, both studies revealed challenges related to procedural standardization and data integration. This approach addresses these issues by unifying force, EMG, and motion under a standardized, synchronized protocol, ensuring consistent contribution of each modality to CI computation.

Although this pilot study focused on the elbow joint, the proposed non-invasive, multimodal system demonstrates high potential for broader clinical and research applications. By integrating inertial measurement units (IMUs), surface electromyography, and force sensors, the system enables three-dimensional motion reconstruction and simultaneous capture of neuromuscular and biomechanical responses. This technological versatility makes it suitable for adaptation to multi-axis joints such as the shoulder, hip, or ankle by recalibrating the axis of movement and adjusting the target muscle groups accordingly.

Several statistical limitations inherent to the small sample size of this pilot study should be acknowledged. With only nine participants, multivariate techniques such as PCA may yield unstable component loadings that are sensitive to individual observations, limiting the generalizability of the extracted latent structure. Similarly, correlation coefficients estimated in small cohorts are susceptible to inflation and may overrepresent the strength of associations, increasing the risk of Type I error. Conversely, the limited sample size also reduces statistical power, increasing the likelihood of Type II errors and the failure to detect smaller but potentially clinically meaningful effects. Although ANOVA results indicated between‑group differences within this dataset, these findings should be interpreted cautiously, as assumption checks are difficult to reliably verify in such small samples. Participant heterogeneity including variability in diagnosis (e.g., cerebral palsy vs. stroke), age (18–42 years), and anthropometric characteristics may have influenced sensor-derived measurements. The results should be interpreted as hypothesis‑generating, motivating future studies with larger, more homogeneous cohorts to enable robust estimation, stable multivariate modeling, and confirmatory statistical testing.

Given the limited sample size and heterogeneous clinical population, these findings should be interpreted as exploratory and hypothesis-generating, providing a foundation for future validation rather than evidence of clinical readiness. Subsequent studies should explicitly evaluate subgroup effects, including etiology (e.g., cerebral palsy, stroke, traumatic brain injury, spinal cord injury) and age category (pediatric versus adult populations), and spasticity level defined by MTS (this study used levels 2–4 only) given known differences in neuromuscular properties and spasticity mechanisms. Longitudinal designs will be essential to assess responsiveness to intervention and test–retest reliability, while larger datasets may enable refinement of weighting strategies, including adaptive or machine‑learning‑based approaches. Importantly, these technologies should also be adapted for pediatric populations, where early and accurate characterization of spasticity is critical.

Future work will focus on formal validation of the proposed Composite Index in larger and more homogeneous cohorts. Based on the observed effect size in this pilot study (η² = 0.68), an estimated sample size of approximately 8–10 participants per spasticity level would be required to achieve 80% statistical power at α = 0.05. This estimate provides an initial benchmark for planning confirmatory studies rather than a definitive requirement. A cross‑sectional validation study designed to (i) assess the stability of Composite Index weights, (ii) evaluate reproducibility across assessors and sessions, and (iii) examine sensitivity across clinically defined spasticity strata would support utility. To reduce heterogeneity, early validation should prioritize single‑etiology cohorts, such as adults with post‑stroke spasticity or individuals with spastic cerebral palsy, before extending to mixed populations. Providing clinicians with quantitative, multimodal assessment tools at the onset of rehabilitation may support more objective treatment planning and improve functional outcomes in children with early-onset motor disorders. Ultimately, the system’s portability, adaptability, and real-time feedback capabilities position it as a promising platform for future widespread clinical use in personalized neurorehabilitation.

## Conclusion

This pilot exploratory study introduces a preliminary multimodal Composite Index for spasticity quantification based on synchronized sEMG, IMU, and force signals acquired during standardized passive stretches. The CI showed internal coherence and preliminary sensitivity to clinical stratification within a small cohort, while enabling separation of reflex and non‑reflex components of hypertonia. Although these results suggest feasibility, they are hypothesis‑generating and not intended to establish clinical validity. Larger, targeted studies are required to confirm robustness, generalizability, and clinical relevance.

## Supporting information

S1 File Dataset. Individual patient data: Composite Index (CI) and Modified Tardieu Scale (MTS) scores.
(XLSX)
